# Real-Time PCR Assay for the Detection of Dermatophytes: Comparison between an In-House Method and a Commercial Kit for the Diagnosis of Dermatophytoses in Patients from Dakar, Senegal

**DOI:** 10.3390/jof7110949

**Published:** 2021-11-10

**Authors:** Jihane Kabtani, Khadim Diongue, Jean-Noël Dione, Anne Delmas, Coralie L’Ollivier, Marie-Claude Amoureux, Daouda Ndiaye, Stéphane Ranque

**Affiliations:** 1 IHU Méditerranée Infection, 13005 Marseille, France; kabtanijihane@hotmail.com (J.K.); jeannoelsaidedione@gmail.com (J.-N.D.); coralie.lollivier@ap-hm.fr (C.L.); 2 Laboratory of Parasitology and Mycology, Aristide le Dantec University Hospital, Dakar 3001, Senegal; khadimase@gmail.com (K.D.); daouda.ndiaye@ucad.edu.sn (D.N.); 3 Service of Parasitology-Mycology, Faculty of Medicine, Pharmacy and Odontology, Cheikh Anta Diop University of Dakar, Dakar 10700, Senegal; 4 Eurobio Scientific, 7, Avenue de Scandinavie Z.A. de Courtabœuf, 91940 Les Ulis, France; anne.rieusset@gmail.com (A.D.); marie-claude.amoureux@univ-amu.fr (M.-C.A.); 5 Campus Timone, Faculté des Sciences Médicales et Paramédicales, Aix-Marseille Université, AP-HM, IRD, SSA, VITROME, 19-21 Boulevard Jean Moulin, 13005 Marseille, France

**Keywords:** dermatophytoses, tinea capitis, tinea unguium, tinea pedis, tinea corporis, real-time PCR, direct ITS sequencing, Africa, Senegal

## Abstract

Background. PCR assays have been developed for the diagnosis of dermatophytes, yet data in African populations are scarce. Objective. This study aimed to compare two PCR assays for the diagnosis of dermatophytosis in outpatients at the Aristide Le Dantec University Hospital in Dakar, Senegal. Patients and methods. A total of 105 samples, including 24 skin, 19 nail and 62 hair samples collected from 99 patients were included in this study. Each sample was subjected to conventional diagnosis (CD), including direct microscopy and culture, and two real-time PCR assays: one in-house (IH)-PCR, used at the University Hospital of Marseille and the Eurobio Scientific commercial kit (CK): designed for the specific detection of six dermatophytes not including *Microsporum audouinii*. Results. Of the 105 specimens, 24.8%, 36.2% and 20% were positive by CD, IH-PCR and CK-PCR, respectively. The IH-PCR and CK-PCR exhibited 88.9% and 65.4% sensitivity, respectively. With a 36.6 diagnostic odd ratio and 1.41 needed to diagnose, the IH-PCR displayed better diagnostic indices than the CK-PCR. It is notable that, when considering the species that it claims to detect, when it came to skin and nail samples, CK-PCR sensitivity increased to 77%. Conclusions. The pan-dermatophyte IH-PCR performed better in the diagnosis of dermatophytosis in this African population than the CK-PCR, which is not designed to detect *M. audouinii*. Nevertheless, both assays exhibited similarly good diagnostic indices for tinea corporis and tinea unguium, both of which are localisations where *M. audouinii* is more rarely involved than in tinea capitis.

## 1. Introduction

Dermatophytoses are superficial mycoses caused by dermatophytes, a group of closely-related fungi that share a special tropism towards keratinised materials for their survival. Dermatophytes infect the stratum corneum (tinea corporis, tinea pedis, etc.), nails (tinea unguium) and hair (tinea capitis) of humans and animals, affecting a huge population worldwide [1].

In Senegal, according to a laboratory-based study conducted on 2026 patients from 2007 to 2011 [2], dermatophytoses were diagnosed in patients aged from 3 months to 89 years, with a mean age of 25.5 years and a 39.3% prevalence. Women were more frequently infected (77%) than men 23%, and the most frequently isolated species was *Trichophyton soudanense* (52.78%).

Conventionally, the laboratory diagnosis of dermatophytosis is based on both direct microscopic examination (DME) of skin, nail or hair samples and cultures [3]. Nevertheless, in a pooled analysis by Levitt et al. [4], the sensitivities of DME (KOH mount) and culture for dermatophytosis diagnosis, were estimated at 73.3% and 41.7%, respectively. In addition, several recurrent problems appear with cultures, such as the increasing isolation of non-dermatophyte filamentous fungi (NDFF), particularly from abnormal nail samples [3]. Therefore, reliable and mostly rapid, non-culture-based diagnostic tools are essential for the detection and identification of dermatophytes as well as for the discrimination of dermatophytes from yeasts and NDFFs.

Polymerase chain reaction (PCR)-based assays for the direct detection and identification of dermatophytes in clinical samples address these challenges. These methods include conventional (end-point) PCR, real-time PCR (either singleplex or multiplex), and PCR-RFLP (restriction fragment length polymorphism) [3,5]. PCR assays have been demonstrated to have higher sensitivity than conventional microscopy and culture methods in patients from Europe [5,6,7], North-America [8], Asia [9] and Australia [10]. In contrast, to the best of our knowledge, dermatophyte PCR diagnostic indices have not yet been evaluated in African patients. Therefore, this study aimed to compare the performance of two real-time PCR assays for the diagnosis of tinea capitis, tinea unguium, tinea pedis and tinea corporis in outpatients who presented with suspected dermatophytosis at the Aristide Le Dantec University Hospital in Dakar, Senegal, one in-house (IH) PCR targeting the 18S rRNA gene and routinely used for the diagnosis of dermatophytoses at Marseille (France) University Hospital, and one commercial kit (CK), EurobioPlex Dermatophytes (Eurobio Scientific), specifically designed for the detection of six dermatophytes (*Trichophyton rubrum*/*violaceum* and *T. soudanense* by homology of sequence, *Trichophyton tonsurans*, *Trichophyton mentagrophytes var. interdigitale*, *Microsporum canis* and *Epidermophyton floccosum*).

## 2. Patients and Methods

### 2.1. Study Design and Sites

**Figure 1:**
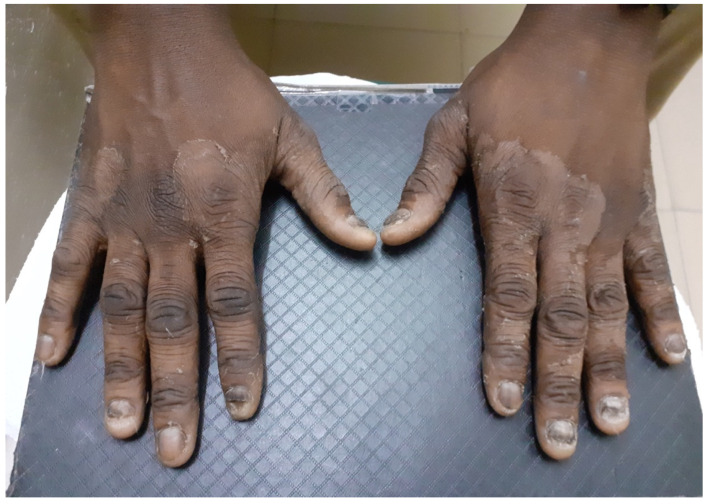
Dermatophytosis of the hands (tinea manuum) and fingernails (tinea unguis).

Between April 2017 and October 2020, patients who attended the Parasitology–Mycology laboratory at the Aristide Le Dantec University Hospital in Dakar, Senegal following a dermatological consultation with suspected dermatophytosis were included in the study. For each patient, the clinical specimens included hair, nail and/or skin samples collected from a dermatophytosis compatible clinical lesion (Figure 1). For tinea capitis, the hair was cut near the root and the skin samples from the scalp were scraped off with a clean scalpel blade at the periphery of the lesions, while pustules were scraped off with a cotton swab. After gently cleaning the affected areas with 70° alcohol, scale samples were collected directly using a scalpel from the edges of the tinea corporis lesions and tinea pedis. For nail samples, affected nails were first cleaned with 70° alcohol and clipped up to the limit between the healthy part and the affected part. The crumbling subungual debris from under the trimmed edge of the nail was then scraped with a scalpel blade. Each collected sample was divided in two. One part was used for the conventional diagnosis in Dakar, and the other was used for PCR-based diagnosis in Marseille. Part of this latter sample intended for PCR was used to repeat the culture in cases where the former part was contaminated at an early stage. For the retrospective evaluation, we selected the samples in sufficient quantities to perform both the conventional methods and the PCR assays. This study was approved by the Ethics Committee of the Cheikh Ant Diop University (0212/2016/CER/UCAD). Informed consent was obtained from the study participants.

### 2.2. Conventional Diagnosis (CD)

Conventional diagnosis was based on both DME and culture, as previously described [11]. DME of all specimens was performed using a 20% KOH solution. All specimens were cultured in two plates/tubes, one containing Sabouraud dextrose agar (SDA) supplemented with chloramphenicol, 0.5 g/L (Bio-Rad, Marnes-la-Coquette, France), and the other containing SDA supplemented with chloramphenicol and cycloheximide, 0.5 g/L (Bio-Rad). Cultures were incubated in an aerobic atmosphere at 22–27 °C and were evaluated for growth after 48 h and then once a week for a month. Isolates were identified based on the growth rate and macroscopic and microscopic characteristics of colonies, and sometimes on biochemical characteristics (urease test) according to the identification key detailed in Chabasse et al. [12]. The conventional dermatophytosis diagnosis was retained when the DME and/or culture were positive.

### 2.3. DNA Extraction

Hair, skin and nail samples were placed into 2 mL tubes containing ceramic beads plus 600 μL of lysis buffer G2 (provided with the Qiagen^TM^ Tissue kit) for mechanical lysis at 6 m/s for 40 s using a FastPrep-24™ Instrument, followed by centrifugation at 10,000× *g* for one minute. The DNA extraction was performed with an EZ1 Advanced XL (Qiagen, les Ulis, France) instrument. A total elution volume of 100 μL of genomic DNA was extracted and stored at −20 °C for future analysis.

### 2.4. In-House PCR Assay

As described in a previous article [5], the real-time PCR was optimised, with a limit of detection of 2 to 3 equivalent dermatophyte genomes per reaction/sample, and run on a CFX96™ (Real-time PCR detection system, Bio-Rad, Marnes-la-Coquette, France) machine. Briefly, the reaction was carried out in a 25 μL volume with 5 μL DNA template, 3.5 μL (20 μm) DMP-F primer (5′-TTATTGCCTCAAACTTCCAT-3′), 3.5 μL (20 μm) DMP-R primer (5′-TAACGAACGAGACCTTAACC-3′), 1 μL (5 μm) DMP labelled probe (5′-FAM-CTAAATAGCCCGGTCGGCGT-3′), and 12 μL of LightCycler™ 480 Probes Master (Roche Diagnostics, Meylan, France). The reaction profile was as follows: 10 min at 95 °C followed by 45 cycles of 10 s at 95 °C; 30 s at 54 °C, 10 s at 72 °C and, finally, one cycle of 30 s at 40 °C. The results were checked with CFX Manager Version 3.1 (Bio-Rad). Positive (*T. rubrum* IHEM 26523 DNA) and non-template controls were included in each run.

### 2.5. Commercial Kit Assay: EurobioPlex Dermatophytes Real-Time PCR

The CK which is under development (Ref; EBX-023, Eurobio Scientific, Les Ulis, France) contains two different OligoMixes that specifically detect different dermatophytes. Each OligoMix contains specific primers and probes; OligoMix 1: *Microsporum canis* (MC), *Trichophyton mentagrophytes*/*interdigitale* (TI) + control of DNA extraction and PCR inhibition (CI-PCR). MC and TI DNA are, respectively, revealed with FAM and HEX labelled probes. OligoMix 2: *Trichophyton rubrum*/*violaceum* (TR/TV; undifferentiated detection, and sometimes *Trichophyton soudanense* due to sequence homology), *Trichophyton tonsurans* (TT) and *Epidermophyton floccosum* (EF) + control of DNA extraction and PCR inhibition (CI-PCR). TR/TV, TT and EF DNA are, respectively, revealed with FAM, HEX and Texas Red labelled probes. Control of DNA extraction and PCR inhibition (CI-PCR) are detected with CY5 label probe. All probes transmit a specific fluorescence succeeding their hydrolysis during the elongation of the sample amplification.

The CK-PCR reaction was carried out in 26 μL volume, with a 5 μL DNA sample in 21 μL Mastermix 1 or 2 (12.5 μL Enzyme, 7.5 μL OligoMix 1 or 2 and 1 μL CI-PCR). Positive controls were supplemented with 5 μL of CP1 (for OligoMix 1) or CP2 (for OligoMix 2) and negative controls with 5 μL of water supplied with the kit (CN-H2O) for both OligoMixes. Each sample was amplified with the two OligoMixes.

The CK-PCR was run on a CFX96™ machine (Real-time PCR detection system, Bio-Rad) and the results were analysed with CFX Manager Version 3.1 (Bio-Rad). The programme performed was as following: three minutes at 95 °C, 40 cycles of amplification for 10 s at 95 °C, 30 s at 56 °C, and 45 s at 72 °C.

### 2.6. ITS Sequencing

Discordant results between CD and/or both PCR assays were further analysed by direct sequencing of the internal transcribed spacers (ITS) of rRNA gene, using universal ITS primers, as previously described [11,13]. Briefly, the reaction was performed in a total volume of 25 μL containing 5 μL of DNA template and 20 μL of PCR-Mix (12.5 μL ATG (AmpliTaq Gold^®^ 360 Master Mix, Applied Biosystems^®^)/6 μL sterile water DNase/RNase free/0.75 μL Forward/Reverse primer). The amplification relied on three PCR reactions for each sample, by using the ITS1/2, ITS3/4 and ITS1/4 primers pairs, respectively (Table 1). Each PCR amplified the ITS1, ITS2 and ITS1-5.8S-ITS2 regions of the rRNA gene, respectively. The PCR program for all PCRs was: 95 °C for 15 min, followed by 39 cycles of 95 °C for 1 min, 56 °C for 30 s, 72 °C for 1 min and a final extension at 72 °C for 5 min. The sequencing reaction was: 96 °C for 1 min, followed by 25 cycles of 96 °C for 10 s, 50 °C for 5 s, 60 °C for 3 min. The sequences were analysed on a 3500 Genetic Analyzer (Applied Biosystems, Inc., Villebon-sur-Yvette, France). The obtained sequences were then assembled and corrected using the CodonCode Aligner software (CodonCode^®^ Corporation, Centerville, MA, USA). The ITS DNA sequences were queried against the GenBank (https://blast.ncbi.nlm.nih.gov/Blast.cgi, accessed on 17 August 2021) via the BLAST (Basic Local Alignment Search Tool), and the MycoBank (http://www.mycobank.org, accessed on 17 August 2021) nucleotide databases (last accessed: 17 August 2021). Identification was retained when the best-match sequences from both databases were concordant. Sequence-based species identification was defined by ≥99% sequence similarity of an ITS rRNA gene region of at least 200 bp length.

### 2.7. Gold Standard (GS)

The dermatophytosis diagnostic gold standard was a positive dermatophyte culture and/or a positive DME. A positive DME is recorded if the culture did not grow a dermatophyte and was retained only if no NDFF was grown from the sample.

### 2.8. Statistical Analysis

Results were presented as frequency tables. The performances of each test, including sensitivity (Se), specificity (Sp), Youden’s index, number needed to diagnose (NND) and diagnostic odds ratio (DOR) were presented. Although not relevant according to our retrospective design, the positive and negative predictive values were calculated for the purpose of comparison with previous studies. A *p* value < 0.05 was considered to be statistically significant. Calculations were performed using the online two-way contingency table analysis (https://statpages.info/ctab2x2.html, accessed on 17 August 2021).

## 3. Results

### 3.1. Characteristics of the Samples

In total, 105 samples (62 hair, 24 skin and 19 nail samples) from 99 patients were included in the study. Of these 99 patients, six provided two different samples, each from distinct clinical lesions. There were 71 (72%) female and 28 (28%) male patients. Patients’ ages ranged from 2 to 80 years with a mean age of 33 (±19) years.

### 3.2. Conventional Methods

**Figure 2:**
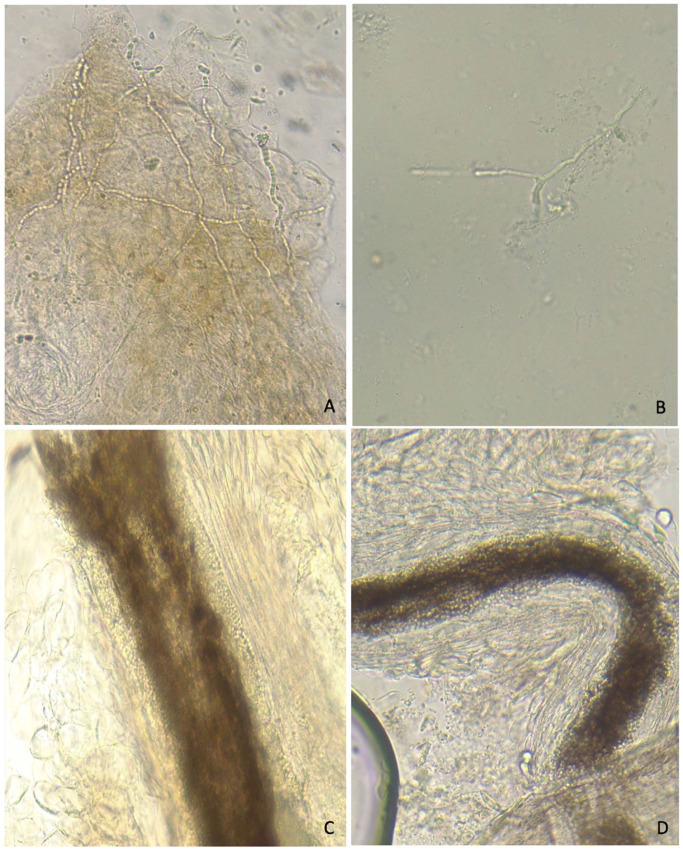
Direct microscopic examination of scales (**top**) or hair (**bottom**) samples. Panel (**A**) dermatophyte-like hyphae formed by chain of arthroconidia; panel (**B**) *Fusarium*-like hyphae with a “dead wood” appearance; panel (**C**) endo-ectothrix hair shaft dermatophyte infection; panel (**D**) endothrix hair shaft dermatophyte infection.

Overall, 26 (24.8%, 95% CI (16.9; 34.1)) samples tested positive for dermatophytes with CD (positive DME and/or culture). DME was positive in 51 samples, of which 27 showed dermatophyte-like hyphae (*n* = 6) and hair involvement (*n* = 21; 17 endothrix and 4 endo-ectothrix lesions). Other positive DME showed *Fusarium*-like hyphae (*n* = 3), budding yeasts (*n* = 16) or budding yeasts associated with hyphae or pseudohyphae (*n* = 5) (Figure 2). All but one of the 27 samples with positive dermatophytes DME yielded a positive dermatophyte culture. Of the 26 positive cultures, six dermatophyte species were identified; *Trichophyton soudanense* was the predominant species. The positive DME and culture results are summarised in Table 1.

An analysis of PCR diagnostic accuracy of both methods of detecting dermatophytes compared to the conventional diagnostics (CD) diagnostic gold standard for detecting dermatophytoses was performed and is presented in a contingency table (Table 2).

### 3.3. In-House (IH) PCR Assay

The IH-PCR was positive for 38 of the 105 samples (36.2%, 95% CI (27.0; 46.1)) of which 24 (63.2%, 95% CI (46.0; 78.2)) were DME positive and 23 (60.5%, 95% CI (43.4; 76.0)) were culture positive. The IH-PCR could detect the five dermatophyte species identified by conventional methods. Of the 38 IH-PCR positive samples, 24 (63.2%) were detected in hair, eight (21%) in skin, and six (15.8%) in nail samples. The IH-PCR detected the presence of non-cultured dermatophytes DNA in 14 (17.9%) of 78 samples with negative conventional methods (DME and culture). The IH-PCR displayed an overall sensitivity of 88.9% and 82% specificity for the diagnosis of dermatophytosis. The other diagnostic indices are summarised in Table 3.

### 3.4. Commercial Kit (CK) EurobioPlex Dermatophytes PCR Assay

The CK-PCR was positive for 21 (20.0%, 95% CI (12.8; 28.9)) of the 105 samples. Of the 21 positive samples, 14 (66.7%, 95% CI (43.0; 85.4)) were both DME and culture positive. The CK could detect the two dermatophytes species, which was expected according to its design, from the panel of collected samples (*T. rubrum* and *soudanense*) among those identified by conventional methods and, as was also expected, it detected no cases of *Microsporum audouinii* infection. Of the 21 CK positive samples, 12 (57%) were detected in hair, five (24%) in skin, and four (19%) in nail samples. The CK detected the presence of non-cultured dermatophytes DNA in seven (9%) of the 78 samples found to be negative using conventional methods (DME and culture). Because the CK-PCR is a specific kit not designed to detect *M. audouinii*, its overall sensitivity was 65.4% for the diagnosis of dermatophytosis on this panel and 95% specificity. It can be best evaluated for its true sensitivity on the two relevant species. On these samples, and after integration of the sequencing results performed on 21 samples to resolve discordant results with CD and/or PCR assays, 26 samples could be included. Twenty were detected and the CK displayed 77% sensitivity. The other diagnostic indices are summarised in Table 3.

### 3.5. Diagnostic Performance According to Sample Type

Regarding the 62 hair samples, the IH-PCR was positive in 24 cases (38.7%, 95% CI (26.6; 51.9)) of which six were negative according to conventional methods, while the CK was positive in 14 cases (22.6%, 95% CI (10.4; 31.4)), with two samples found to be negative by conventional methods. Therefore, on hair samples, compared to conventional methods as the gold standard, the IH and the CK-PCRs displayed 85.7% and 57.1% sensitivity, respectively (which was to be expected for the latter due to the non-detection of four *M. audouinii* positive samples, which is not a target of the kit). After integration of the sequencing results, and considering the specific targets of the CK, 17 dermatophyte hair samples were expected to be detectable by CK. In total, 82.4% were indeed detected. IH-PCR and CK displayed 85.4% and 92.7% specificity, respectively (Table 4).

Of the 24 skin samples, the IH method was positive in eight (33.3%, 95% CI (15.6; 55.3)) samples, five of which were negative by conventional methods, whereas the CK was positive on five (20.8%, 95% CI (7.1; 42.2)) samples, three of which were negative by conventional methods. The IH method and the CK displayed a sensitivity of 100%, and a 73% and 86% specificity on skin samples, respectively. The other diagnostic indices of these methods are summarised in Table 5. Nevertheless, as shown in Table 7, according to the ITS direct sequencing arbitration, the IH-PCR presented four FP with no FN on skin samples, while the CK-PCR displayed three FP and three FN results.

Regarding the 19 nails samples, the IH method was positive in six (31.6%, 95% CI (12.6; 56.6)) cases of which half were negative by conventional methods, whereas the CK was positive in four (21.1%, 95% CI (6.1; 45.6)) samples with only one sample found negative by conventional methods. Both IH and CK methods displayed a sensitivity of 100% (Table 6); there were three FP with the IH-PCR and only one FP with the CK-PCR on nail samples.

Further analysis of the discordant PCR results by direct ITS sequencing as included in Table 7.

For instance, on hair samples, we found four false positive (FP) and three false negative (FN) with the IH-PCR, whereas there were no FP and five FN (among which three anticipated FN due to *M. audouinii* infections) with the CK-PCR (Table 7).

## 4. Discussion

This study compared the performances of two real-time PCR assays for the detection of dermatophytes in samples from patients in Dakar, Senegal who presented with a clinical suspicion of dermatophytosis. The samples analysed were hair, skin and nail samples. This sample diversity is one strength of our study, in contrast with many others, which were limited only to nail samples [7,8,14] or, at best, to nail and skin samples [9,10]. The IH real-time PCR assay routinely used at the University Hospital of Marseille, France is designed to detect the main clinical dermatophyte species, but gives no indication as to the species of the dermatophyte species that was detected in the sample [5]. The CK uses two distinct OligoMixes that detect specific dermatophyte species and, thus, gives some indication as to which dermatophyte species is present in the sample. Notably, the first mix detects *Microsporum canis*, *Trichophyton mentagrophytes* and *T. interdigitale*, whereas the second mix detects *T. rubrum*, *T. violaceum*, *T. soudanense*, *T. tonsurans* and *Epidermophyton floccosum*. This capacity of the CK to indicate the dermatophyte species involved is useful and may potentially influence the patients’ treatment, particularly in those with tinea capitis. Indeed, as we have previously mentioned [11], tinea capitis requires specific treatment for several reasons that have been detailed in Gräser et al. [15]. Firstly, these authors pointed out epidemiological circumstances increasing the risk of reinfection. For example, *M. canis* is commonly transmitted by an animal source, while *Nannizzia gypsea* (*M. gypseum*), which causes similar lesions, is transmitted by contact with contaminated soil. The source of infection identification is important for the prevention of reinfection and secondary cases. Secondly, they pointed out the optimum treatment regimen, which differs depending on the dermatophyte species involved, e.g., *T. tonsurans* tinea capitis usually requires shorter treatment regimens than *M. canis* tinea capitis, which is, to some extent, able to evade drug exposure by forming arthroconidia outside the hair shaft. Thirdly, they pointed out the importance of distinguishing dermatophytes from pseudo-dermatophyte fungi, which cause dermatophytosis-like infections, especially onychomycoses, but are resistant to the usual anti-dermatophyte drugs.

Several commercial systems using molecular-based methods for the detection of dermatophytes that cover different species spectra have been developed. The majority of them do not discriminate zoophilic from anthropophilic species, which is important in order to efficiently treat the sources of infection as explained above [15,16]. Likewise, a large number of in-house PCR techniques have been developed for the direct detection of dermatophytes. Some of these tests, particularly those based on real-time PCR display interesting features but none is standardized [16]. In this context, we conducted this study and chose the EurobioPlex Dermatophytes (Eurobio Scientific) CK, which is specifically designed for the detection and identification of six dermatophyte species, including *T. soudanense*, which is the most frequent species in Senegal, and compared this CK with the in-house method, validated and routinely used at the University Hospital of Marseille (France) [5].

As is the case for most of the PCR-based methods aiming to directly detect dermatophyte DNA in clinical samples, these two methods were designed and validated on European patient samples [5]. Similar methods are also available in other parts of the world [8,9,10] but, to the best of our knowledge, none have yet been validated in Africa.

In sum, in these African patient samples, with a higher sensitivity, a higher DOR and a lower NND, the IH method seems to display better diagnostic indices than the CK method, despite the excellent 91% specificity of this latter method, which is better than the 82.1% specificity of the IH method. The results for the CK are nevertheless to be expected since it was designed with the goal of differentiating between specific dermatophyte species, most of which are not present in this panel of samples. Nevertheless, both tests are effective, displaying 63.2% and 66.7% PPV for the IH and the CK-PCR, respectively. However, these performances are lower than the 90% PPV found on skin and nail samples by Ross et al. using a commercial multiplex tandem PCR assay [10]. With respect to the IH method, in the present study the diagnostic indices were enhanced compared to the 79.1% sensitivity, 73.1% specificity, 10.3 DOR and 1.9 NND found in the initial clinical evaluation study [5].

With regards to hair samples, the relatively low overall sensitivity of the CK is very likely due to the fact that the CK is not designed to detect *M. audouinii*. However, it should be noted that only two cases of *M. audouinii* were missed by the CK-PCR, in addition to which eight other false negative cases were noted. This could be due to the scarce quantity of hair collected for mycological analysis [17] and might explain why Ohst et al. [18] found a higher sensitivity of 90.4% but a lower specificity of 55.1% on hair samples.

It should be noted that both PCR methods displayed 100% sensitivity on skin and nail samples, and the CK-PCR displayed slightly higher specificity, NND and DOR than the IH-PCR. The performances of the two methods on nail samples were enhanced when compared to the 80% sensitivity and 74.4% specificity of the DermaGenius^TM^ Nail real-time PCR assay [7], and the 87.5% sensitivity and 85% specificity of real-time PCR targeting the β-tubulin gene, developed in Iran [19]. Similar results were found by Gong et al. [20] on nail samples using a real-time PCR for pan-dermatophyte detection with 92.4% sensitivity and 97.3% specificity.

Whereas both PCR assays displayed 75% PPV on hair samples, the CK showed higher performance than the IH-PCR (40% vs. 25% PPV) on skin samples and (75% vs. 50% PPV) on nail samples. These two dermatophyte PCR assays performed better on hair samples than the one used in a study conducted in Sweden, which had a 71% PPV [6]. In contrast, with a β-tubulin-targeted real-time PCR, Motamedi et al. [19] found a higher 78.1% PPV on skin and similar performances (PPV = 56.5%) on nail samples. Gong et al. [20] found a 97.2% PPV on nail samples. These better performances on nail samples could be explained by the fact that more abundant material is usually collected from onychomycosis compared to hair or skin. Therefore, the high sensitivity of PCR assays for the diagnosis of onychomycoses might be due to the relatively higher quantity of DNA extracted from nails compared to other clinical samples.

Another strength of our study was to further analyse the discrepant results of each diagnostic technique by direct ITS sequencing. The real-time PCR positive samples in which the direct ITS sequencing was negative, because it either did not succeed or identified a non-dermatophyte fungus, were classified as FP. We acknowledge the limitation of this interpretation because these negative direct sequencing results may be due to the expected lower analytical sensitivity of the direct ITS sequencing compared to real-time PCR assays or to mixed infections involving dermatophytes and yeast or other filamentous fungi in which the non-dermatophyte DNA has been preferentially amplified and detected. For all these reasons, we may have overestimated the PCR FP rate. In contrast to culture based methods, another global limitation of this qPCR approach is its inadequacy for the follow-up of dermatophytosis treatment outcome because it cannot distinguish DNA originating from live or dead fungi.

## 5. Conclusions

In conclusion, both PCR assays exhibited good indices for the diagnosis of dermatophytosis on both skin and nails samples, both of which are localisations where *M. audouinii* is relatively less frequently involved than in tinea capitis. In this respect, the CK-PCR was limited by its inability by design to detect *M. audouinii*. This dermatophyte species is mostly involved in tinea capitis but can also infect the skin and nails, especially in populations where this species has a relatively high prevalence. The IH-PCR assay appears to be better suited than CK-PCR for the diagnosis of dermatophytosis in African populations, such as the one in this study conducted in Senegal. More generally, the two methods can be considered complementary for the diagnosis of dermatophytoses by PCR.

## Figures and Tables

**Table 1:** Positive results of direct microscopic examination and culture (*n* = 105).

Direct Microscopic Examination (*n* = 51)		Hair (*n* = 62)	Skin (*n* = 24)	Nail (*n* = 19)
		21 (33.87)	19 (79.2)	11 (57.89)
**Culture** **(*n* = 45)**	**Dermatophytes (*n* = 26)**			
	*T. soudanense*	11	0	1
	*M. audouinii* *	4	0	0
	*T. mentagrophytes*	3	0	1
	*T. rubrum*	3	1	1
	*T. interdigitale*	0	1	0
	**Yeasts (*n* = 15)**			
	*C. albicans*	0	3	3
	*Non-albicans Candida*	0	4	3
	*Trichosporon* sp.	0	1	1
	**Non-dermatophyte filamentous fungi (*n* = 4)**			
	*Fusarium* sp.	0	2	0
	*Fusarium* sp./*C. albicans*	0	1	1

* not included in the design of the EurobioPlex Dermatophytes kit.

**Table 2:** Contingency table of the in-house (IH)- and commercial kit (CK)-PCR results compared to the diagnostic gold standard for the detection of dermatophytoses.

		IH-PCR		CK-PCR		
		Positive	Negative	Positive	Negative	Total
Gold Standard	Positive	23	3	17	9 *	26
	Negative	15	64	4	75	79
	Total	38	67	21	84	105

* 4 are *M. audouinii* not included in the CK-PCR detection panel.

**Table 3:** Overall diagnostic indices of the IH and CK-PCR assays compared to conventional methods as the diagnostic gold standard.

Parameters	IH	95% CI	CK	95% CI
Sensitivity	0.89	[0.73–0.97]	0.65/(0.77 *)	[0.47–0.84]
Specificity	0.82	[0.77–0.85]	0.95	[0.90–0.99]
PPV	0.63	[0.52–0.69]	0.85	[0.70–1.0]
NPV	0.96	[0.89–0.998]	0.88	[0.81–0.95]
Youden’s index	0.71	[0.49–0.82]	0.43	[0.21–0.60]
NND	1.41	[1.22–2.03]	2.33	[1.67–4.68]
DOR	36.57	[8.67–179.04]	10.92	[3.29–37.63]

* sensitivity on species targeted by CK after sequence confirmation. IH: in-house PCR; CK: commercial kit PCR; PPV: Positive predictive value; NPV: Negative predictive value; NND: Number need to diagnose; DOR: Diagnostic odd ratio.

**Table 4:** Diagnostic indices of the IH and CK-PCR assays on (*n* = 62) hair samples compared to conventional methods as the diagnostic gold standard.

	IH	95% CI	CK	95% CI
Sensitivity	0.86	[0.68–0.96]	0.57 (0.82 *)	[0.36–0.78]
Specificity	0.85	[0.76–0.91]	0.93	[0.84–0.98]
PPV	0.75	[0.59–0.84]	0.75	[0.46–0.93]
NPV	0.92	[0.82–0.98]	0.76	[0.69–0.80]
Youden’s index	0.71	[0.44–0.86]	0.36	[0.10–0.51]
NND	1.41	[1.16–2.29]	2.81	[1.96–9.62]
DOR	35.0	[6.63–218.02]	9.50	[1.89–53.92]

* sensitivity on species targeted by CK after sequence confirmation.

**Table 5:** Diagnostic indices of the IH and CK-PCR assays on (*n* = 24) skin samples compared to conventional methods as the gold standard.

	IH	95% CI	CK	95% CI
Sensitivity	1.00	[0.21–1.00]	1.00	[0.210–1.00]
Specificity	0.73	[0.66–0.73]	0.86	[0.79–0.86]
PPV	0.25	[0.05–0.25]	0.40	[0.08–0.40]
NPV	1.00	[0.90–1.00]	1.00	[0.92–1.00]
Youden’s index	0.73	[−0.14–0.73]	0.73	[−0.15–0.73]
NND	1.385	[1.38–7.23]	1.16	[1.16–611.70]
DOR	+∞	[0.50–+∞]	+∞	[1.01–+∞]

**Table 6:** Diagnostic indices of the IH and CK-PCR assays on (*n* = 19) nail samples compared to conventional methods as the gold standard.

	IH	95% CI	CK	95% CI
Sensitivity	1.00	[0.34–1.00]	1.00	[0.36–1.00]
Specificity	0.81	[0.69–0.81]	0.94	[0.82–0.94]
PPV	0.50	[0.17–0.50]	0.750	[0.27–0.75]
NPV	1.00	[0.85–1.00]	1.00	[0.87–1.00]
Youden’s index	0.81	[0.03–0.81]	0.94	[0.17–0.94]
NND	1.23	[1.2–31.49]	1.07	[1.07–5.80]
DOR	1.155	[−∞–+∞]	+∞	[2.46–+∞]

**Table 7:** False positive and negative cases of the in-house (IH) and the EurobioPlex (CK) PCR assays according to direct ITS sequencing results (reading from left to right).

**Hair** **(*n* = 62)**	IH (+) = 24	CD (+) = 18		FP = 4
		CD (−) = 6	Seq (+) = 2	
			Seq (−) = 4	
	IH (−) = 38	CD (−) = 35		FN = 3
		CD (+) = 3		
	CK (+) = 14	CD (+) = 12		FP = 0
		CD (−) = 2	Seq (+) = 2	
			Seq (−) = 0	
	CK (−) = 48	CD (−) = 41		FN = 5 *
		CD (+) = 7	Seq (−) = 5	
			Seq (+) = 2	
**Skin** **(*n* = 24)**	IH (+) = 9	CD (+) = 2		FP = 4
		CD (−) = 7	Seq (+) = 3	
			Seq (−) = 4	
	IH (−) = 15	CD (−) = 15		FN = 0
		CD (+) = 0		
	CK (+) = 4	CD (+) = 2		FP = 1
		CD (−) = 2	Seq (+) = 1	
			Seq (−) = 1	
	CK (−) = 20	CD (−) = 16		FN = 2
		CD (+) = 4	Seq (−) = 2	
			Seq (+) = 2	
**Nails** **(*n* = 19)**	IH (+) = 6	CD (+) = 3		FP = 3
		CD (−) = 3	Seq (+) = 0	
			Seq (−) = 3	
	IH (−) = 13	CD (−) = 13		FN = 0
		CD (+) = 0		
	CK (+) = 3	CD (+) = 3		FP = 0
		CD (−) = 0		
	CK (−) = 16	CD (−) = 16		FN = 0
		CD (+) = 0		

IH: in-house (method); CK: commercial kit; CD: conventional method; Seq: direct ITS sequencing; FP: false positive; FN: false negative; (+): positive; (−): negative; * two *Microsporum audouinii* isolates as expected.

## Data Availability

The data presented in this study are openly available in the IHU Méditerranée Infection data repository at [https://doi.org/10.35081/dcrt-ms93], reference [https://www.mediterranee-infection.com/acces-ressources/donnees-pour-articles/data-from-real-time-pcr-assay-for-the-detection-of-dermatophytes].
